# Evaluating a Group‐Based Intervention Addressing Fear of Childbirth in Multiparous Pregnant Women: A Mixed Methods Feasibility Study

**DOI:** 10.1111/jan.17073

**Published:** 2025-05-23

**Authors:** Laura Sandström, Marja Kaunonen, Heini Huhtala, Anna Liisa Aho

**Affiliations:** ^1^ Tampere University Tampere Finland; ^2^ Wellbeing Services County of Pirkanmaa Tampere Finland

**Keywords:** fear of childbirth, feasibility study, group‐based intervention, mixed methods, multiparous women, pregnancy

## Abstract

**Aim:**

To assess the feasibility and acceptability of a group‐based intervention for addressing fear of childbirth in multiparous women.

**Design:**

Single‐arm non‐randomised feasibility trial with a convergent mixed methods design.

**Methods:**

The intervention, conducted at a central maternity hospital and led by a psychiatric nurse and a midwife, included three prenatal and one postnatal face‐to‐face group sessions, supplemented by a phone call. Quantitative measures were gathered via self‐report questionnaires at baseline, before birth, and post‐intervention. Exit interviews were conducted with participants and interventionists. Additional data included records from recruiting midwives, the primary investigator, and diaries filled out by the interventionists after each session. Primary outcomes assessed included recruitment, adherence, acceptability, and fidelity. Secondary outcomes included fear of childbirth, anxiety, depression, and childbirth experience.

**Results:**

The intervention was feasible and acceptable. Recruitment and adherence aligned with pre‐study expectations. Indicative results suggested potential improvement in fear of childbirth and helped secure a positive childbirth experience, particularly through peer discussions and the birthing class.

**Conclusion:**

While the intervention is considered feasible and acceptable, it requires further refinement before proceeding to a multicentre randomised controlled pilot trial.

**Implications for Patient Care:**

The group‐based intervention may have potential in reducing fear of childbirth and enhancing the childbirth experience for multiparous women. These women may particularly benefit from peer support and childbirth classes.

**Impact:**

Rising fear of childbirth can adversely affect mothers, families, and society. Existing interventions often target primiparous women, neglecting multiparous women. This study evaluated a novel group‐based intervention for fear of childbirth in multiparous women in Finland. Findings confirmed its feasibility and acceptability, with preliminary results showing a positive impact on fear of childbirth. Further research is needed to validate these findings. This research has implications for multiparous women and the healthcare professionals supporting them.

**Reporting Methods:**

The study adhered to CONSORT extension guidelines for reporting randomised pilot and feasibility trials (Supplementary file 1) and the TIDieR checklist (Supplementary file 2).

**Patient Contribution:**

Limited patient and public involvement was incorporated, focusing on the development of the intervention.

**Trial Registration:** clinicaltrials.gov: NCT05766202

**Summary**

- What does this paper contribute to the wider clinical community?
  ○ Initial findings indicate that a group‐based intervention is feasible and acceptable to implement in a central maternity hospital setting and may be beneficial in reducing fear of childbirth in multiparous women.
  ○ Childbirth classes and peer support should be considered for multiparous women experiencing fear of childbirth.
  ○ Multidisciplinary collaboration between psychiatry and midwifery is important in addressing fear of childbirth.

## 1 Introduction

Due to the increasing prevalence of fear of childbirth (Sanjari et al. 2022) and its potential long‐term adverse effects (Dencker et al. 2019), there has been significant interest in developing targeted interventions. Despite active research in the field of fear of childbirth, intervention studies tailored specifically for multiparous women are less developed. Most studies have focused on pregnant primiparas (O'Connell et al. 2021), even though the prevalence of fear is similar in both groups (Sanjari et al. 2022). Effective interventions for primiparas may not be feasible or acceptable for multiparas, as the aetiology of fear differs. Multiparas are often fearful due to previous negative or traumatic birth experiences, while primiparas fear the unknown (Dencker et al. 2019).

Another shortcoming in previous intervention research of fear of childbirth is the lack of feasibility study publications. Without examining and reporting feasibility and acceptability, refinement, further testing, or implementation of complex interventions might fail, leading to research waste (Bowen et al. 2009; Skivington et al. 2021). Additionally, published feasibility studies addressing fear of childbirth have focused on cause‐and‐effect results (Nieminen et al. 2016; Byrne et al. 2014), which are not relevant in studies with limited statistical power (Bowen et al. 2009).

To address these shortcomings and improve care for multiparous women experiencing fear of childbirth, we developed the Multiparas Overcoming Childbirth Fear Through Intervention and Empowerment (MOTIVE). This study evaluates the feasibility and acceptability of the MOTIVE intervention to determine its suitability for a larger‐scale clinical trial.

## 2 Background

According to a systematic review and meta‐analysis, the global prevalence of severe fear of childbirth was 16% and it seems to have increased during the past decade (Sanjari et al. 2022). A recent concept analysis defines fear of childbirth as a pregnant woman exhibiting cognitive and affective disorders, somatic symptoms, and behavioural changes when facing actual or potential harm from pregnancy and childbirth (Chen et al. 2024). While pain is a common source of fear, concerns also include potential injuries to the child or oneself, complications, or interventions such as emergency caesarean sections (Wigert et al. 2020). Fear of childbirth can result in adverse consequences not only for the woman and her overall well‐being but also her baby and the whole family. It may lead to increased requests for caesarean section or other medical interventions, negatively impacting the birth experience (Dencker et al. 2019; Viirman et al. 2022). Additionally, fear of childbirth is associated with postpartum depression, post‐traumatic stress disorder, and the need for psychiatric care (Dencker et al. 2019). In countries with low birth rates, such as Finland, fear of childbirth can be a significant factor as it may deter women from having multiple children despite their desire to do so (Dencker et al. 2019). Consequently, fear of childbirth affects not only the individual but also society as a whole.

In Finland, maternity care reaches almost 100% of pregnant women and is publicly funded. Primary care, provided by public health nurses, is the main setting for discussing thoughts and fears about childbirth. If fear of childbirth is detected, women can be referred to outpatient clinics, typically located in maternity hospitals, where midwives and obstetricians offer counselling. However, to our knowledge, there is no evidence supporting the effectiveness of this counselling for treating fear of childbirth. Additionally, various forms of psychological support are available, but the type and accessibility of treatment vary significantly depending on the woman's location. Moreover, primiparous women receive more treatment options than multiparous women, who are limited to outpatient clinics.

## 3 The Study

The current study investigated the feasibility and acceptability of the MOTIVE intervention in a central maternity hospital setting. As per the study protocol (Sandström et al. 2024), feasibility refers to measuring recruitment, attendance, and retention rates, along with reasons for declining participation or withdrawing from the trial. The acceptability of the design, contents, and delivery, as well as the fidelity of the intervention sessions, were assessed. Lastly, secondary data measures, including fear of childbirth, anxiety, depression, and childbirth experience, were evaluated to help estimate the initial efficacy of the intervention.

## 4 Methods

### 4.1 Design

This was a single‐arm non‐randomised feasibility study. We used a convergent mixed methods research approach, gathering and integrating both quantitative and qualitative data. This resulted in an interpretation grounded in the combined strengths of both data sets (Creswell and Plano Clark 2018). We adhere to pragmatism as our philosophical stance, as pragmatic research answers questions about how an intervention can be used in actual real‐life settings (Creswell and Plano Clark 2018), which was also the aim of this study.

### 4.2 Study Setting and Sampling

Recruitment occurred at a single maternity outpatient clinic in Finland from April 2023 to January 2024. Midwives discussed the study with potential participants during routine 20‐week ultrasound scans. The trial was also advertised via posters in the clinic and on social media, allowing women to self‐refer by contacting the first author for eligibility confirmation.

Given the feasibility nature of this study, no formal sample size calculations were conducted. Instead, the sample size was pragmatically determined based on patient flow at the recruitment site. The maternity outpatient clinic receives approximately 30 multiparous women for their 20‐week ultrasound scan each month, totaling 360 over the 12‐month recruitment period. Based on registry data of fear of childbirth diagnoses in multiparous women in Finland, it was estimated that 15% would meet the inclusion criteria (THL 2024), resulting in 54 eligible participants. Previous recruitment for similar interventions for women with fear of childbirth indicates a 30% volunteer rate (Toohill et al. 2014). Thus, the aim was to recruit 16 participants within 12 months, in accordance with guidelines for pilot and feasibility studies that recommend sample sizes of 12 to 24 participants (Billingham et al. 2013). This target was confirmed in consultation with a statistician.

### 4.3 Inclusion and Exclusion Criteria

The inclusion criteria comprised multiparous women under 35 weeks of pregnancy at the intervention's onset, aged over 18 years, understand Finnish, with self‐reported fear of childbirth, and are willing to participate in the study. Exclusion criteria included being openly psychotic, at risk of suicide, or having a serious substance abuse problem. Eligible women sent a signed consent form to the first author, who then informed them of their group's meeting times.

### 4.4 Intervention

The overall approach of the entire study from design to the trial phases is guided by the United Kingdom Medical Research Council (MRC) framework for developing and evaluating complex interventions (Skivington et al. 2021). MOTIVE was iteratively developed by integrating insights from previous intervention studies (O'Connell et al. 2021) and qualitative inquiries, where multiparous women shared their experiences and desired professional support for addressing their fear of childbirth (Sandström et al. 2023, 2022). A logic model was developed to monitor intervention fidelity and provide insight into how the intervention did or did not work in practice, identify any unintended consequences, and refine the design of the future trial (Supplementary file 3).

The primary objective of MOTIVE is to support pregnant multiparous women experiencing fear of childbirth, with the aim of alleviating fear. Sessions were conducted by a psychiatric nurse and midwife at a central maternity hospital every Wednesday afternoon for two hours. The interventionists were provided with an informational package for each session that they followed. MOTIVE comprised four group sessions, three held prenatally and one postnatally, with each group accommodating up to eight participants. Additionally, a postnatal phone call was incorporated, allowing the psychiatric nurse to discuss the birth one‐on‐one with the participant before the final group session.

**FIGURE 1:**
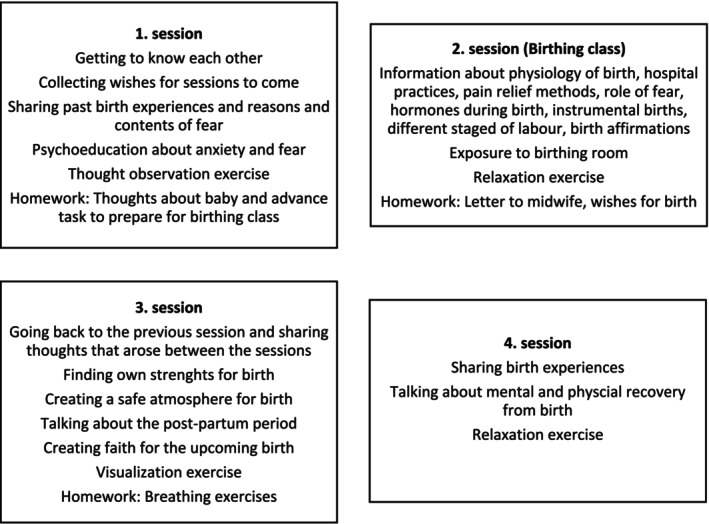
Contents of sessions of MOTIVE.

The first group session was held at pregnancy week 32 (±3 weeks), with the second and third sessions approximately two weeks apart, and the final session four to eight weeks post‐birth. Each session covered specific core topics, but the content was tailored to the group's needs. The topics covered in the sessions are detailed in Figure 1. Participants were treated as active learners through individual and group work, emphasising sharing experiences, feelings, and thoughts, and incorporating homework between sessions. The first group started in August 2023 and the last group finished in May 2024.

### 4.5 Data Collection and Measures

#### 4.5.1 Quantitative Data

Midwives recruiting multiparous women during ultrasound scans kept records of recruitment rates. The first author also documented women who self‐recruited through posters at the maternity outpatient clinic or via social media advertisements.

Participants completed questionnaires at three points: baseline, pre‐birth (four weeks post‐baseline), and post‐intervention (after birth up to 16 weeks post‐baseline). The questionnaires covered sociodemographic characteristics, medical information (mental health, pregnancy, and birth), content of fear of childbirth (measured on a Likert scale from 1 to 5), previous healthcare experiences, session attendance, and clinical outcomes (Table 1). Participants also evaluated MOTIVE using a Likert scale from 1 (strongly disagree) to 5 (strongly agree) and graded the intervention from 4 (very low satisfaction) to 10 (very high satisfaction). Time was allocated during each session to complete the questionnaires. If absent, the questionnaire was sent to the participant's home.

**TABLE 1:** Outcome measures.

Concept	Assessment	Time‐points	Possible range	Interpretation	Cronbach *α*
Fear of childbirth	FOBS	All three	0–100	≥ 60 points indicator of childbirth fear	0.91
Pregnancy related anxiety	PRAQ‐R2	Baseline and before birth	10–50	Higher score indicates higher anxiety	0.85
Anxiety	HADS‐A	All three	0–21	No anxiety ≤ 7 points	0.89–0.93
				Anxiety of clinical significance 8–10 points	
				Severe anxiety 11–21 points	
Depression	EPDS	All three	0–30	No depression ≤ 12 points	0.87
				Mild 13–14 points	
				Moderate 15–18 points	
				Major ≥ 19 points	
Birth experience	CPS	Post‐intervention	12–48	Higher score indicates more negative perception of both delivery and the first postpartum week	0.82
	VAS	Baseline and post‐intervention	0–10	0 very negative experience	Not applicable
				10 very positive experience	

Abbreviations: CPS, Childbirth Perception Scale (Truijens et al. 2014); EPDS, Edinburgh Postnatal Depression Scale (Cox et al. 1987); FOBS, Fear of Childbirth Scale (Haines et al. 2011); HADS‐A, Hospital Anxiety and Depression Scale, the anxiety subscale (Zigmond and Snaith 1983); PRAQ‐R2, Pregnancy Related Anxiety Questionnaire Revised (Huizink et al. 2016); VAS, Visual Analog Scale (Joensuu et al. 2021).

#### 4.5.2 Qualitative Data

Qualitative data was gathered to assess acceptability and fidelity of the intervention. All participants were contacted by the first author for a semi‐structured individual interview to gather their views and experiences regarding the MOTIVE intervention. A total of 15 participants were interviewed, with 13 conducted face‐to‐face and 2 via telephone, based on participant preference, within two weeks after the last intervention session. The interviews lasted approximately 39 min (range: 22–51 min) and were audio recorded.

In addition to collecting qualitative data from participants, data was also gathered from the psychiatric nurse and midwife who facilitated the intervention. They participated in a semi‐structured pair interview in June 2024. The interview was audio recorded and lasted 80 min. The interventionists were also asked to keep a diary after each session, noting observations about group dynamics, their feelings and thoughts, any surprises, and whether the session went as planned, and if not, why.

### 4.6 Analysis

#### 4.6.1 Quantitative Analysis

Quantitative data regarding demographics and outcomes relating to recruitment and adherence were analysed and reported using descriptive statistics. Likert‐scale responses of acceptability were reported using medians, with minimum and maximum values. Due to the small sample size and non‐normal data distribution for all clinical outcomes, we presented medians and ranges alongside means with standard deviations to ensure comparability with previous studies. Repeated clinical outcomes (fear of childbirth, anxiety and depression) were plotted graphically per participant to assess change across the three time points.

#### 4.6.2 Qualitative Analysis

All interviews were transcribed verbatim by the first author. Qualitative data concerning acceptance were analysed using thematic analysis with a deductive approach, as outlined by Braun and Clarke (2006), while remaining open to emergent themes. Coding was guided by key constructs of the TIDieR guideline (Hoffmann et al. 2014) to ensure comprehensive inquiry and understanding of relevant intervention factors. Qualitative data concerning fidelity were analysed also with thematic analysis, but with an inductive approach (Braun and Clarke 2006). Transcripts were sorted, coded, and categorised to facilitate thematic analysis. Themes were refined multiple times, continuing until each theme was fully developed and illustrated with quotes from the data set. Atlas.ti (version 24) was used to facilitate the analysis.

#### 4.6.3 Integration of Data Sets

Following the completion of the quantitative and qualitative data analysis concerning acceptance, a side‐by‐side comparison was done to examine (dis)congruence of the findings. We created a joint display of quantitative and qualitative findings and resulting meta‐inference to help gain a deeper understanding of the acceptance of the intervention (Guetterman et al. 2015).

### 4.7 Ethical Considerations

The trial was registered before recruitment commenced (NCT05766202). Ethics approval was obtained from Pirkanmaa Wellbeing Services County's Regional Research Ethics Committee (R22124H/14.2.2023), and study approval was granted by Kanta Häme Central Hospital (HVA/1275/13.00.01/2023).

Potential participants were reminded by recruiting midwives that participation was voluntary, would not affect their normal care pathways, and could be discontinued at any time. Personal information was handled confidentially by the first author, with a data management plan ensuring safe data collection and maintenance. Written informed consent was obtained from all participants, who had the opportunity to ask questions before consenting. They were also given the first author's contact information for any queries. All names used in this paper are pseudonyms.

## 5 Results

### 5.1 Participants, Recruitment and Adherence

**FIGURE 2:**
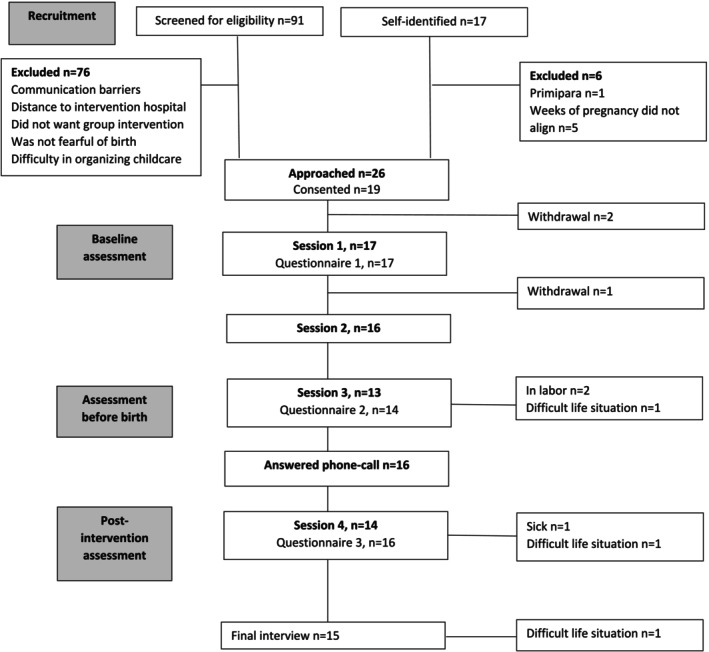
CONSORT flow diagram of the participants.

The feasibility and acceptability assessment criteria and results are detailed in Table 2. In total, 91 individuals were screened at the routine week 20 ultrasound scan. Fifteen women (16.5%) met the inclusion criteria. In addition, 17 women self‐identified as potential participants through poster advertisements or social media, with eleven (64.7%) meeting inclusion criteria. Ultimately, 19 individuals completed the consent form, and recruitment concluded after 10 months, surpassing the target of 16 participants. Of these 19 participants, eight (42.1%) were recruited by the midwives, two (10.5%) from the posters at the outpatient clinic at the hospital, and nine (47.4%) from social media. Four groups were assembled. Four participants were recruited for the first and second groups, six for the third group, and five for the last group (Figure 2).

**TABLE 2:** Feasibility and acceptability assessment criteria and results.

Objective	Progression criteria	Results
*Feasibility*		
To evaluate the screening rate	360 women are screened for eligibility criteria at the routine week 20 ultrasound scan during the 12‐month recruitment period	91 women were screened for eligibility criteria at the routine week 20 ultrasound scan over a period of 10 months
To evaluate the proportion of women meeting eligibility criteria	54 women are eligible to participate in the intervention during the 12‐month recruitment period	26 women were eligible to participate in the intervention over a period of 10 months
To evaluate the proportion of eligible women consenting to participate in the study	16 eligible women are willing to consent to participate in the study within the 12‐month recruitment period	19 women consented to participate in the study over a period of 10 months
To evaluate the proportion of participants taking part in all four intervention sessions and answering the call after birth	≥ 80% of participants who begin the intervention take part in all four sessions and answer the phone call after birth	100% took part in the first session
		94.1% took part in the second session
		76.5% took part in the third session
		94.1% answered the phone‐call
		82.4% took part in the fourth session
To evaluate the proportion of participants completing all three questionnaires and participating in the exit interview	≥ 80% of participants that begin the intervention complete all three questionnaires and participate in the exit interview	100% completed the first questionnaire
		82.4% completed the second questionnaire
		94.1% completed the third questionnaire
		88.2% participated in the exit interview
*Acceptability*		
To assess the acceptability of the intervention	The trial will be considered feasible if the participants and the interventionists find the intervention acceptable	Participants and interventionists found the intervention acceptable
	No adverse events are allowed	No adverse events were reported

Attendance logs showed that per session on average 88.2% of the women participated. Reasons for non‐attendance were illness, labour, and family‐related circumstances. For retention, all but one participant completed the questionnaires from baseline to post‐intervention, resulting in a 94.1% (*n* = 16) retention rate. Baseline data was available from 17 participants. Most participants were expecting their second child and began the intervention at 31 weeks of pregnancy. Nearly half had previously been diagnosed with a mental health disorder, primarily anxiety and depression. Over half had experienced fear of childbirth in a previous pregnancy (Table 3). The five most feared objects listed in the questionnaire were caesarean section, difficult recovery, pain, and injury to the baby or themselves.

**TABLE 3:** Participant characteristics at baseline (*n* = 17) and birth outcomes post‐intervention (*n* = 16).

	Women starting the intervention (*n* = 17)	
	*n*/(mean)	%/(standard deviation, SD)
Age in years	(30)	(5.3)
Married or living with a partner	17	100
Education		
Secondary education	6	35.3
Bachelor's degree	4	23.5
Master's degree or higher	7	41.2
Employment		
Full‐time	7	41.2
Part‐time	2	11.8
Self‐employed	1	5.9
Unemployed	1	5.9
Student	1	5.9
Stay‐at‐home mother	5	29.4
Previous mental health diagnosis	7	41.2
Gestational age (weeks)	(31)	(2.3)
Uncomplicated pregnancy	14	82.4
Number of previous births		
One	14	82.4
Two or more	3	17.6
Fear of childbirth in previous pregnancy	9	52.9
Preferred mode of delivery		
Vaginal	16	94.1
Caesarean section	1	5.9

	Women finishing the intervention (*n* = 16)	
	*n*/mean/median	%/(SD)/min, max
Weeks of pregnancy at labour	39.2	(1.0)
Onset of labour		
Spontaneous	7	43.8
Induction	7	43.8
Caesarean	2	12.5
Pain relief during labour		
Opioid injection	2	12.5
Nitrous oxide	10	62.5
Paracervical block	4	25.0
Epidural	11	68.8
Spinal	5	31.3
Pudendal block	11	68.8
Delivery outcome		
Vaginal	11	68.8
Vacuum extraction	1	6.3
Breech	1	6.3
Elective caesarean	2	12.5
Emergency caesarean	1	6.3
Duration of labour (hours)	7.5	0, 25
Apgar score at 1 min	9.5	6, 10
Perceived support from primary care		
Poor	2	12.5
Moderate	7	43.8
Good	7	43.8
Perceived support from fear of childbirth clinics		
Moderate	2	20
Good	8	80

The retention rate for the second questionnaire was 82.4% (*n* = 14). Post‐intervention data was collected from 16 participants. All participants' pregnancies were full‐term, with nearly half of the births induced and medical pain relief used in all births. Two participants had elective caesareans, one due to fear of childbirth. Over half (*n* = 10) received additional specialised care for fear of childbirth, with support perceived as moderate or good (Table 3). The retention rate for interviews was 88.2% (*n* = 15).

Drop‐out was low throughout the whole intervention: only three participants (15.8%) withdrew from the study, two due to family‐related situations (death of a close family member, unable to get a nanny for the first‐born), and the third one was not reached; thus, the reason for drop‐out is unknown.

### 5.2 Intervention Acceptability

Table 4 presents a joint display of qualitative findings on acceptability alongside mean acceptability scores from the questionnaire divided into four pre‐determined themes of overall acceptance “The intervention exceeded expectations”, design “The intervention design was appealing and participant‐friendly”, content “The intervention contents alleviated fears and prepared for the upcoming birth”, and delivery “The intervention delivery well‐suited needs and life situation” of the intervention followed by eight sub‐themes.

**TABLE 4:** Joint display of acceptability of MOTIVE.

Acceptability	Theme	Sub‐theme	Questionnaire claim	Median	Range^a^	Meta‐inference
Overall	The intervention exceeded expectations		Overall score for the intervention	9	8–10	The intervention was well received and reached its goal in alleviating fear of childbirth
			I would recommend the intervention to other multiparous women suffering from fear of childbirth	5	4–5^b^	
				**Median**	**Range** ^b^	
Design	The intervention design was appealing and participant‐friendly	Coming together with multiparous women experiencing fear of childbirth fostered a sense of solidarity	I appreciated the group‐based nature of the intervention	5	3–5	The group format was an asset, as it encouraged participants to share and discuss together. The trial was designed to require minimal effort from participants
			I received peer‐support	5	1–5	
			The group maintained a respectful atmosphere	5	4–5	
		Effortless and approachable facilitation style	Enrolling in the trial was easy	5	4–5	
			The reminder messages were helpful	5	4–5	
			The questionnaires were easy to fill in during the sessions	5	4–5	
Content delivery	The intervention contents alleviated fears and prepared for the upcoming birth		Topics covered were useful	4.5	3–5	The content formed a cohesive whole, which was found to be helpful in alleviating fear and preparing for birth.
		Discussions with peers provided perspective	Sharing experiences with peers was useful	5	2–5	
		Gaining knowledge about childbirth created a sense of control.	The birthing class was useful	5	1–5	
			Taking part in the intervention helped me prepare for birth	5	2–5	
			Participating in the intervention gave me the confidence I needed when going to give birth	4	1–5	
		Learning coping strategies for anxiety and fear promoted relaxation.	The relaxation exercises were useful	4	1–5	
			I learned new ways to manage my emotions	4.5	2–5	
	The intervention delivery well‐suited needs and life situation	The right people	The midwife and psychiatric nurse were the right professionals to deliver the intervention	5	4–5	Four face‐to‐face sessions in a relaxed environment, along with a phone call with empathetic and knowledgeable professionals, were found to be acceptable
			The call after birth was useful	5	3–5	
		The right place	The location of the sessions was convenient for me	5	3–5	
			Visiting the birthing room was useless	1	1–4	
		The right time	The sessions fit my schedule	5	4–5	
			I would have wished that the sessions would have started earlier on in pregnancy	3	1–5	

^a^ 4 = worst possible score, 10 = best possible score.

^b^ 1 = totally disagree, 5 = totally agree.

#### 5.2.1 The Intervention Exceeded Expectations

Participants rated the intervention highly, with all (*n* = 16) recommending it to other multiparous women experiencing fear of childbirth, indicating high satisfaction with MOTIVE. Almost all participants (88%, *n* = 14) indicated that the intervention helped alleviate their fear. They reported that fear was no longer a pressing issue, as the birth had been a positive experience. Interventionists also found the intervention well received and meaningful, believing participants benefited from it.

Participating really helped me, and I think it would be very important to continue offering this to multiparous women in the future. Lotta, group 1 (G1)

#### 5.2.2 The Intervention Design Was Appealing and Participant‐Friendly

##### 5.2.2.1 Coming Together With Multiparous Women Experiencing Fear of Childbirth Fostered a Sense of Solidarity

Participants valued the group‐based nature of the intervention, with peer support being a primary reason for enrolment. Indeed, most participants (81%, *n* = 13) reported receiving peer support. Hearing that they were not alone in having a bad previous experience and subsequent fear was important and provided a sense of belonging and understanding. Being part of the group fostered a sense of solidarity.

I wouldn't have even believed how much, somehow, it felt like we are in this together. Helvi, G2

It was nice to just sit and chat, listen to others' thoughts, and share my own. There was a social benefit, as usually in mother groups or WhatsApp groups, people might not want to bring up their fears as much. We try to stay positive in those other groups, so it was nice that this group was specifically established with that topic in mind. Selma, G1

However, the formation of groups and peer support was not straightforward in all groups. Shyer participants were initially apprehensive about speaking to strangers but found the courage to share due to the group's permissive and respectful atmosphere. Participants could share as much or as little as they wished, with no pressure to contribute. Conversely, more talkative participants struggled to find their place in the group, as they found it challenging to balance speaking and allowing others space to share. This imbalance prevented them from receiving the peer support they sought and led to frustration when quieter participants did not engage, causing them to hold back.

I'm quite talkative, so it was a bit like… The others were maybe a bit subdued. There was one other person who was also a bit more talkative, but it felt like… I was afraid of taking up space from others. Yeah, I tried to hold back a bit, because when something was asked, no one really started saying anything. Maybe we were just so different. And I completely understand that it's not so easy to suddenly open up or something. So, the conversations remained superficial. Rea, G3

##### 5.2.2.2 Effortless and Approachable Facilitation Style

Regarding inclusion criteria, both participants and interventionists felt it was important to target only multiparous women for two reasons. First, public maternity care did not offer them any group‐based treatment. Second, for peer support to succeed, it was crucial that all participants had at least one childbirth experience. Additionally, the interventionists stated that having a subjective experience of fear of childbirth was sufficient; there was no need to implement a cut‐off for fear for inclusion criteria. Participants agreed that it was beneficial to hear from women with different levels and types of fears. However, one participant mentioned she could not have participated if someone had experienced stillbirth or neonatal death, as it would have been too distressing. Also, an interventionist suggested excluding multiparas with unprocessed or recent traumatic experiences. Despite these concerns, no harms or unintended effects were reported by participants.

What caught my attention was that this group was for multiparous women because usually when you see something like that, it's all for first‐time mothers. Cecilia (G4)

Enrolling in the trial was easy and required minimal effort, making participants feel welcomed. Participants were sent text message reminders a few days before their group sessions, which helped them orientate towards the upcoming session. Participants' attitudes towards filling out the questionnaires were neutral, and they found it convenient to complete them during the sessions.

The reminder messages were good. Yeah, they reminded me that the session was coming up and what we would cover that time. They also reminded me where I must be and at what time, which was useful information. Elviira, G4

The interventionists struggled without prior information or notes about the participants, making it difficult to recall who shared what. They proposed that participants write a letter detailing their fears for the interventionists to read before the first session. Additionally, keeping session memos would help alleviate their cognitive burden.

Yes, you always had to think about who was sitting where and connect that to the discussions, as there were no notes like you would usually have, such as visit records. It did feel mentally taxing, as you had to rely heavily on memory. At one point, there were three overlapping groups, which made it even more confusing to keep track of everyone's issues. Interventionist 1

#### 5.2.3 The Intervention Contents Alleviated Fears and Prepared for the Upcoming Birth

##### 5.2.3.1 Discussions With Peers Provided Perspective

Indeed, 94% of participants stated that the topics covered during the intervention were useful. Participants found discussions with peers particularly helpful. Hearing about others' fears and sharing previous birth experiences provided perspective on their own feelings and experiences. Processing fears and facing them in a supportive group normalised their feelings. The interventionists emphasised the importance of discussing fear as an emotion, educating participants about fear of childbirth and its potential consequences for hormonal regulation during birth.

Or, in a way, I realized, ‘Well, these are just fears.’ Everyone has different situations, and everyone has their own fears, but somehow hearing about other peoples' fears helped me to take some distance from my own thoughts. Selma, G1

##### 5.2.3.2 Gaining Knowledge About Childbirth Created a Sense of Control

Second, despite being multiparous women with at least one previous birth experience, some participants expressed that they lacked sufficient information or had incorrect knowledge about birth or pain relief methods. This was because several participants had given birth to their first child during the COVID‐19 pandemic and had not attended a birthing class at all. Moreover, many expressed that when attending a birth class with their first‐born, they did not know what to ask, and the information could not be put into context since they had no experience of what was to come. Additionally, for some, the first birth had taken place two decades ago in a different hospital, so their knowledge was not up to date.

Hence, the participants valued the opportunity to attend a birth class now and expressed that it was, in addition to peer support, a very important part of the intervention. Participants stated that without this birthing class, they would not have paused to thoughtfully consider the upcoming birth and their wishes for it. Participating together with their partners gave them a sense of fellowship and shared preparation. The class provided them with knowledge of their options, although participants expressed a desire for more information about caesarean sections. Nonetheless, they learned new things about the physiology of birth, different pain relief methods, and their options. They wrote a birth plan, which gave them agency in their own birth and overall helped them feel secure and confident. Participants especially valued the applied knowledge they were provided with and appreciated that their needs and questions were taken into consideration.

And of course, I really liked that there was an actual childbirth class. When I was pregnant last time during Covid‐19, there were no classes. So, both my partner and I got a lot of new information, even though we've already been through one birth. For example, I had no idea how the baby rotates inside before coming out; it really has to make quite a few turns. Sofia, G1

##### 5.2.3.3 Learning Coping Strategies for Anxiety and Fear Promoted Relaxation

Lastly, in the interviews, participants mentioned that they used various relaxation techniques, affirmations, and breathing exercises from the sessions in their daily lives during pregnancy when anxiety, fear, or panic arose. Although some admitted that relaxation exercises were not their thing and did not enjoy this part of the intervention, they did make use of breathing exercises during childbirth. Both women who gave birth vaginally and those who had a caesarean section utilised the exercises.

No, I don't like them (breathing exercises). I fundamentally don't like them. Every time they said we were going to do a breathing exercise; I just rolled my eyes. But I did recall the breathing exercises during the actual event, during the C‐section. Sara G3

#### 5.2.4 The Intervention Delivery Well‐Suited Needs and Life Situation

##### 5.2.4.1 The Right People

Both the psychiatric nurse and midwife were described as perfect to facilitate the intervention, with one covering the psychological and the other covering tangible aspects of fear and birth. The interventionists were described as empathic and knowledgeable. Participants especially appreciated the role of the interventionists as moderators of the discussions, providing a safe space to share intimate thoughts, feelings, and past experiences, where difficult emotions were addressed together.

They were wonderful. It was nice to have two instructors with different perspectives rather than two who were the same. One of them brought a deeper understanding of the mind and its aspects, while the other one provided practical knowledge from the delivery room. This way, we got a bit of both sides. After all, childbirth is both a clinical procedure and a significant emotional event. Selma, G1

Empathy is crucial in these situations, and their (interventionists) facial expressions and non‐verbal communication were very important to me. I'm very sensitive to non‐verbal cues, and both interventionists were excellent at this. Helmi, G3

The interventionists were dedicated to helping the participants with their fears. They became attached to the participants, and the last sessions with each group were described as wistful. The interventionists appreciated each other's expertise and found that they could complement each other, as both had their own favourite subjects. They also mentioned that personal chemistry between the interventionists was important. Since they knew each other and had worked together previously, they felt that their collaboration was seamless, often able to finish each other's sentences.

I thought it worked well because we had already worked together before, so that was a plus. Interventionist 1

You know what the other is talking about. Interventionist 2

The phone call after birth where the participants had the opportunity to talk to the psychiatric nurse one‐on‐one was relevant to the participants. It enabled them to talk about their experience with a familiar professional. The call helped them revisit the birth with guidance and support, forming a complete picture of what had happened.

Yeah, it was good because it allowed me to go through the birth and think about the whole experience. This time, the whole birth experience kind of got left behind because the baby had challenges with (the baby's) weight gain at first. There was all that pumping, supplementing, and breastfeeding chaos, so the whole birth experience got overshadowed by that. Going through it on the phone helped me process it. Anna, G3

##### 5.2.4.2 The Right Place

Some participants faced a commute of over an hour to reach the hospital where the sessions were held. However, this was not perceived as a problem. In fact, participants valued their own time away from home and its responsibilities. The participants expressed that taking part in the sessions was the highlight of their week, as they could focus only on themselves and the upcoming birth. Without these face‐to‐face sessions, participants would not have taken the time to process their fears or express their wishes for the birth. None of them preferred video meetings or digital support.

It was me time. I could fully focus on myself and on the upcoming. Asta, G2

The room where the sessions were held was described as calm and comfortable, almost homelike which promoted the participants' sense of security.

I thought it was good. The building where the sessions were held is not so hospital‐like. Because if we had all sat in the delivery ward meeting room, it wouldn't have been as comfortable. It's nicer to sit on sofas and not have it feel so clinical. Airi, G1

The second session, where participants could visit the birthing room and familiarise themselves with different pain relief methods and birthing positions along with their partners, was well‐received. Seeing the birthing room in a calm situation changed their perception of it as a horrible place. However, many participants felt that also seeing the operating room would have been helpful, not just for those with an elective caesarean section.

Yes, it was really useful when we visited the delivery room. It wasn't some kind of monster anymore, like the room was somewhere out there. I had been in the delivery room with our first‐born, but I had no memory of it. So, it was nice to see the place. I had thought it was some kind of special room, but it was just an ordinary room! Alma G2

##### 5.2.4.3 The Right Time

All sessions were held on Wednesday afternoons from 4 pm to 6 pm. Participants stated that this timing worked well for them, and since they were informed well in advance, it was relatively easy to arrange babysitters or have their spouse care for the children at home after work. The interventionists found it quite easy to implement the sessions into their work schedule since they were given the freedom to plan their own working hours.

It was… I managed to fit it in. I always managed to arrange care, and then my husband would pick (our child) up from daycare after work, so it worked out well. Ruusa G2

Some participants eagerly awaited the intervention and would have preferred it to start earlier in pregnancy. The interventionists agreed that this could be beneficial. They suggested the first session should be held at 20 weeks of pregnancy or later. Starting earlier might not be beneficial as they wouldn't be psychologically ready to think about the birth, and there is a higher concern about miscarriage in the first and second trimesters. However, most participants felt that four group sessions were sufficient and practical for their schedules.

Yes, the sessions were at the right time. But I guess it wouldn't have hurt to have one more session. Cecilia, G4

### 5.3 Intervention Fidelity

Fidelity of delivery was assessed through diaries and interviews with both interventionists and participants. Two main themes were identified: sessions were structured based on the interventionists' growing experience and participants' needs, and some planned content was entirely omitted.

#### 5.3.1 Sessions Were Structured Based on the Interventionists' Growing Experience and Participants' Needs

Interventionists reported that facilitating groups became easier over time, gaining confidence to tailor sessions individually. The first group served as a pilot, with material rushed through. As they progressed, sessions became more participant‐driven, with the fourth group feeling routine. Interviews revealed that the foundational training conducted prior to the commencement of the intervention enhanced interventionists' competence and confidence. However, they expressed a preference for the training to be held closer to the first session to improve content retention.

In its own way, a rhythm had already developed by the time it reached the fourth group. Interventionist 2

Sessions were tailored to participants' needs, with each group presenting unique fears and experiences, leading to varied discussions and knowledge needs. Interventionists emphasised the importance of treating each participant as an individual within the group to ensure they felt secure.

And then since each group was different, the discussions were also different in a way. It always went according to the needs of the group, where we would stop more and where they wanted the discussion to go. Sharing together. I thought it worked well. Interventionist 1

#### 5.3.2 Some Planned Content Was Entirely Omitted

Due to time constraints, some planned content was not covered. The two‐hour sessions proved insufficient as participants actively engaged in discussions. This happened consistently in every group, with the same content being left out. Extending all the sessions to three hours was deemed impractical and burdensome. Therefore, both the interventionists and most participants suggested adding an extra session to the intervention. This one additional session was unanimously suggested to be placed at the beginning of the intervention and for the focus to be only on getting to know each other, reasons for participating, sharing own experiences and fears. In addition, the last session could have lasted three hours, to enable everybody enough time to share their birth experience.

We talked with the other mothers too about how it would have been nice to stay here longer, as it somehow feels like the two hours always went by quickly. Lotta, group 1

Also, certain intervention activities requiring functionality or self‐direction were impractical. Homework was often neglected due to participants' busy lives with toddlers, lack of time, or energy. Exercises were sometimes perceived as too difficult or unhelpful. For instance, thinking about their baby was challenging for many; one participant avoided it, fearing the baby wouldn't survive childbirth. Breathing exercises were also unpopular and not practiced at home by all. Nevertheless, writing a letter to the midwife was well‐received, and all but one participant wrote a birthing letter.

Well, I never remembered to do them (homework), so they were always just in my bag the next time I went to the session. Anna group 2

The inclusive part of the birthing class didn't go entirely as planned. Participants were hesitant to try different birthing positions or drug‐free pain relief methods (e.g., gua‐sha, transcutaneous electrical nerve stimulation “TENS”) with their partners. Interventionists suggested that these exercises might be more feasible if couples could try them behind curtains for privacy. Additionally, not all spouses attended due to childcare issues.

Maybe it was the part in the birthing room. I don't know if it was because there were others. You probably thought they would try all the positions, but they didn't. Interventionist 2

### 5.4 Clinical Outcomes

**FIGURE 3:**
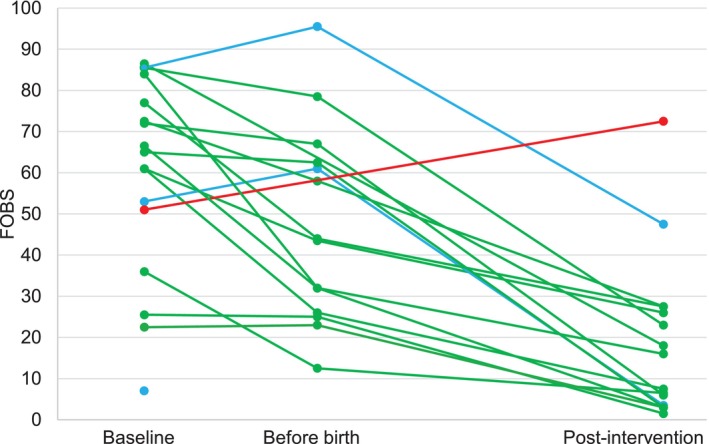
Outcome measures of fear of childbirth per participant at baseline, before birth and post‐intervention.

**FIGURE 4:**
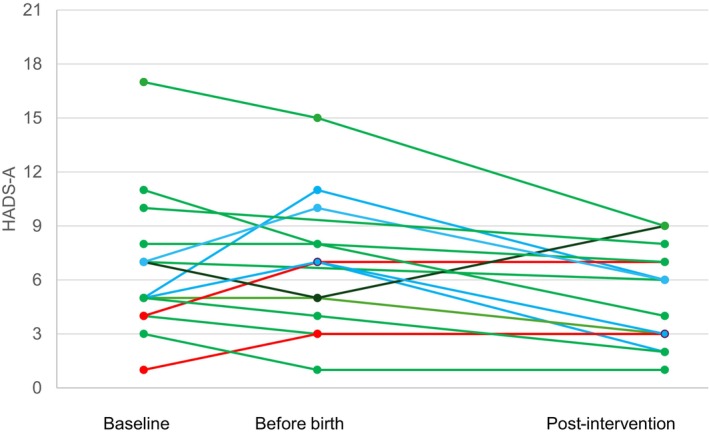
Outcome measures of anxiety per participant at baseline, before birth and post‐intervention.

**FIGURE 5:**
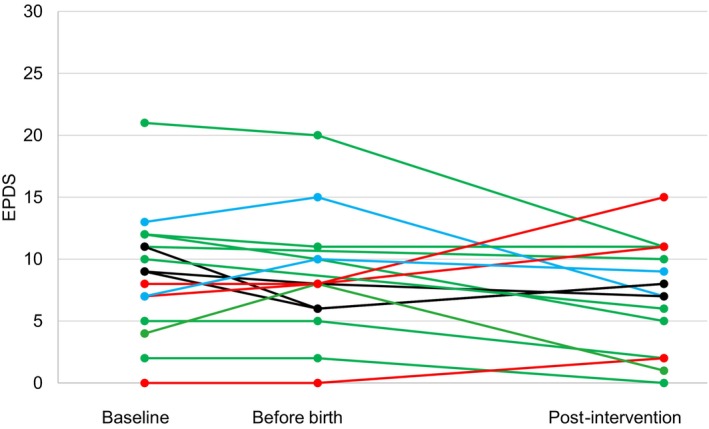
Outcome measures of depression per participant at baseline, before birth and post‐intervention.

In Table 5 we present the mean scores and standard deviations for each outcome measure at different measurement points. In addition, in Figures 3, 4, 5 changes in fear of childbirth, anxiety, and depression are presented per participant.

**TABLE 5:** Outcome measures for fear of childbirth, anxiety, depression, and childbirth experience at baseline, before birth, and post‐intervention, mean and standard deviation (SD).

Scale	Outcome	Baseline *n* = 17		Before birth *n* = 14		Post‐intervention *n* = 16	
		Mean	SD	Mean	SD	Mean	SD
FOBS	Fear of childbirth	59.4	24.1	47.2	24.0	18.3	19.4
HADS‐A	Anxiety	6.4	3.7	6.7	3.7	4.9	2.6
PRAQ‐R2	Pregnancy related anxiety						
	Fear of giving birth	3.2	0.8	3.0	1.0		
	Worries about bearing a physically or mentally handicapped child	2.7	0.9	2.5	1.0		
	Concern about own appearance	2.9	1.2	2.8	1.1		
	Total	29.0	6.5	27.7	5.8		
EPDS	Depression	8.5	4.9	8.4	5.0	7.1	4.2
CPS	Childbirth experience					22.7	4.5
VAS	Childbirth experience	5.9^a^	2.7			8.2	1.0

^a^ Evaluation of previous birth experience.

At baseline, the median fear sum score was 65 (range 7–86.5). At the before‐birth measurement, the median fear sum score decreased to 44 (range 12.5–95.5), and at post‐intervention continued to decrease to 11.8 (range 1.5–72.5). When examining the changes in fear of childbirth per participant from Figure 3, most are green, which indicates that fear declined between measurement points. Only one participant's fear increased between the assessment points (red line). She was also the only one to score over 60 points, indicating childbirth fear at post‐intervention. In addition, two participants experienced a peak in fear levels before birth, which then declined after birth (blue lines).

For anxiety, two (12%) of the participants had severe anxiety at baseline according to HADS‐A with the median sum scores being 5 (range 1–7). At the before birth measurement, there was a slight increase in anxiety, but the median remained within the ‘no anxiety’ range according to the HADS‐A scale classification (median 7, range 1–15). Post‐intervention, no participants experienced severe anxiety, with the median sum scores decreasing to 5 (range 1–9). For anxiety over half of the participants (56.3%) had an even decline of anxiety over time (green lines), but for some anxiety peaked before birth, and then declined post‐intervention (blue lines). For two participants anxiety increased over time (red line), and for one anxiety decreased before birth but increased at the last measurement (black line) (Figure 4). Similarly, pregnancy‐related anxiety was moderate at baseline (median 29, range 18–39) and remained at the same level before birth (median 28, range 19–38). Not surprisingly, the subscale fear of giving birth was the highest source of anxiety during pregnancy (Table 5).

As for depression, at baseline, one participant (5.9%) had severe depression, while the median sum score was 9 (range 0–21), indicating no depression. At the before birth measurement, the median depression sum score slightly decreased to 8 (range 0–20). Post‐intervention, no participants suffered from severe depression, with the median sum score being 7.5 (range 0–15). When examining changes per participant from Figure 5, for most participants, sum scores of depression decreased between measurement points (green line), but there were also those who had higher depression scores post‐intervention (red lines) than at baseline, or who peaked or plummeted before birth (blue and black lines).

Medians of childbirth experiences post‐intervention, measured with both CPS and VAS, were 21 (range 17–32) and 8 (range 6.5–10) respectively, indicating a positive experience, especially when compared to the baseline questionnaire, where participants reported their previous experience as being less positive (median 6, range 1–9) (Table 5).

## 6 Discussion

### 6.1 Feasibility, Acceptability and Fidelity

Midwives screened 91 individuals instead of the intended 300 over the ten months. The failure to recruit might be due to the midwives' inadequate training and experience in recruitment, not perceiving recruitment or the intervention as meaningful, or not having enough time to tell the women at the ultrasound scans about the ongoing study (Briel et al. 2021). This issue must be addressed before advancing to the next stage of the trial. One potential solution is to conduct a multicenter trial, which would allow for a larger proportion of women to be screened. In addition, more emphasis must be placed on the training of midwives involved in recruitment.

Although the screening rates at the ultrasound scans fell short of our initial goals, we successfully reached our target sample size of sixteen participants under the planned 12‐month timeframe. Successful recruitment can be attributed to several factors. These include: a realistic recruitment goal within the planned timeframe, social media as a supplementary recruitment channel to face‐to‐face recruitment, broad enough inclusion criteria, the participants finding the intervention attractive and potentially beneficial, participation not being considered too strenuous, and the first author overseeing the whole process, from training to recruiting and being actively in touch with the recruitment site.

Based on feedback from participants and interventionists, the exclusion criteria will be altered. Women who have experienced stillbirth or neonatal death, or other recent traumatic events will be excluded due to the potential risk of vicarious traumatization in a group‐based intervention. Nonetheless, this group of women cannot be overlooked. Early, intensive, and structured trauma‐focused interventions for persistent symptoms may be beneficial, but further research is needed (Jomeen et al. 2025).

Attendance was robust, with over 80% of participants completing all four sessions and responding to the post‐birth phone call, meeting the protocol's objective (Sandström et al. 2024). However, attendance for the third session fell below the target due to one participant withdrawing, two participants being in labour, and one participant experiencing a challenging personal situation. Nonetheless, we employed strategies which likely contributed to overall high attendance rates (Robinson et al. 2015). These included minimising participant burden, the trial being categorised and perceived as low risk, choosing a convenient location for sessions, the adequate number of sessions, hiring culturally competent staff with strong interpersonal skills to facilitate the intervention, and reminding participants of sessions a few days in advance. In addition, the group‐based nature fostered a sense of solidarity and belonging among the participants, which in turn acted as a social motivator, encouraging them to continue attending. The knowledgeable and empathic psychiatric nurse and midwife also played a crucial role in ensuring participants attended sessions. It proved important to have two types of expertise: knowledge of psychological processes and knowledge of childbirth.

As for data collection, completion was acceptable and met the criteria for successful progression. Results from a systematic review and meta‐analysis suggest that strategies that aim to reduce participant burden (e.g., flexibility in data collection methods) might be most effective in maximising retention (Teague et al. 2018). When participants were unable to attend the sessions where questionnaires were filled out, sending the questionnaires home for them to complete and return to the researcher proved to be an effective complementary approach.

Both participants and interventionists found that the contents of the intervention met the needs of women experiencing fear of childbirth. First, especially peer support where they were able to share their previous experiences and feelings with the support of the interventionists proved to be important to participants. Similar findings regarding the benefits of peer support have been documented in previous studies (O'Connell et al. 2021). However, to enable the interventionists to support the participants in the best possible way, they should write records of every session to help them recall the different stories of participants. Additionally, incorporating a letter where the participant writes their fear of childbirth story upon registration could be useful. It would encourage the participant to start actively processing their fear before the first session, and it would help the interventionists become familiar with the participants beforehand and better understand their needs. Second, in Finland, multiparous women are not offered birthing classes, and overall, there has been a shift to providing remote classes where the woman and her partner do not have the opportunity to see the birthing room or meet up with a midwife. Hence, the birthing class, and especially the part where they had the opportunity to visit the birthing room with their partners, proved to be meaningful. Nevertheless, based on the results, some changes, such as a tour of the operating room and a private space to try out different birthing positions and non‐pharmacological pain relief methods should be incorporated into the birthing class. In addition, it could be helpful for the entire birthing class to be held in the birthing room, with a focus on practical aspects and the needs of participants. Based on the findings, birthing classes for multiparous women should be incorporated into maternity care, as gaining information may help many mitigate their fears (Wigert et al. 2020).

In response to fidelity findings, a more fluid and flexible approach to delivery will be obtained. As intended, the focus was on encouraging meaningful discussions and sharing experiences opposed to sessions strictly focused on the material. Interviews indicate that multiparous women benefit from moderated peer discussions. This should remain a key focus of future interventions. Nevertheless, the participant‐focused approach should be emphasised in the foundational training more so that interventionists do not feel pressured to go through all the material. Additionally, an extra session could be incorporated at the beginning of the intervention, focusing solely on getting to know each other. This could enhance the peer support and the sense of belonging that some participants felt was lacking.

Homework will not be left out even though all participants did not do their homework, but it will be included as an option for those who find it appealing and helpful. In addition, the last session after birth will be extended to three hours (including a break) and should focus only on sharing the past birth experience and leave out content in relation to self‐management, feeding, sleep, and nutrition.

### 6.2 Secondary Outcomes

As for the clinical outcomes, the results indicate that the level of fear of childbirth declined as the intervention proceeded. This result aligns with previous feasibility studies that used similar strategies for women with fear of childbirth, such as psychoeducation, mindfulness, prenatal education, cognitive‐behavioural therapy or acceptance and commitment therapy (Nieminen et al. 2016; Byrne et al. 2014). Although fear of childbirth appeared to decrease during the study period, a similar trend was not as evident for anxiety and depression. This might be due to the relatively low baseline measurements of anxiety and depression.

Nevertheless, we cannot be certain that the decrease in fear of childbirth was solely due to the intervention, as this was not a randomised controlled trial. Participants received both standard and specialised care for fear of childbirth alongside the MOTIVE intervention, with substantial variation in the level of support they experienced from these services, which might have influenced their fear. Additionally, the decrease in fear of childbirth might be attributed to the normal psychological processes that occur at different stages of pregnancy and after birth (Hildingsson et al. 2011). Therefore, the low level of fear of childbirth after birth might result from a combination of treatment alongside MOTIVE, the relief of having the birth behind them, and a positive birth experience, rather than the intervention itself. However, in the interviews, participants mentioned that participating in MOTIVE helped them with their fears. Therefore, the role of the intervention in alleviating fears should not be overlooked.

Regarding measurement of birth experience, according to protocol (Sandström et al. 2024) we used two scales in the post‐intervention questionnaire: the CPS and VAS. In general, participants evaluated their birth experiences as good or very good at post‐intervention. This positive evaluation might have influenced the level of fear at the post‐intervention assessment, as we know that negative experiences often lead to elevated levels of fear of childbirth (Dencker et al. 2019). Additionally, most of the participants were pregnant with their second child, which might have affected their birth experience. It is known that poor childbirth experiences are often associated with interventions and adverse outcomes, which are more common in first births (Taheri et al. 2018; Chauhan et al. 2020). Nevertheless, the birthing class was particularly beneficial in preparing for childbirth according to the interviews with participants, and they learned new things that they used during birth. Thus, participating in MOTIVE might have helped them secure a positive birth experience.

### 6.3 Strengths and Limitations

There are several strengths to this study. Firstly, we adhered to the study protocol that was previously published (Sandström et al. 2024). Secondly, the trial was prospectively registered on ClinicalTrials.gov, which enhances the transparency of our trial and helps prevent publication bias and selective reporting (Aslam et al. 2013). Additionally, due to precise reporting, the trial methods and findings may be generalisable to similar settings as those in this study. Thirdly, using a theoretical framework such as the Medical Research Council, which considers the complexity of the design and contextual interactions, enhances the intervention's efficiency and user experience (Skivington et al. 2021). Lastly, the mixed methods design can be considered a strength, as the use of both quantitative and qualitative data provides a more comprehensive overview of the intervention, its feasibility, and its possible impact on participants' fear (Creswell and Plano Clark 2018). In addition, collecting data from both participants and interventionists provided a broad understanding of different views on the intervention.

Our study, however, is not without limitations. The first limitation was the use of the FOBS scale. Given the simple nature of the scale, we conducted a double translation, and the questionnaires were piloted prior to data collection, resulting in no changes to FOBS. However, FOBS remains unvalidated in Finland. This limitation will need to be addressed before conducting a definitive trial. Second, even though sending questionnaires home to those participants who could not participate in the sessions where questionnaires were filled increased retention rates, it might have an impact on the results because these participants filled out the questionnaires in different settings and slightly differing time points. Third, while we predefined progression criteria for feasibility results, it would have been preferable to use a flexible approach, analogous to a red/amber/green traffic light system, rather than a simple stop/go basis (Avery et al. 2017). Drawing from this error, we will incorporate the traffic light system in the subsequent phase of the trial. Fourth, the study used a nonrandomised design carried out at a single site—this limits the understanding of randomisation and recruitment. Fifth, the small sample size limits the range of perspectives, especially from those who did not adhere to participating in all sessions or dropped out. Lastly, as this study did not include a control group, it is possible that the observed changes would have happened regardless of the intervention. Although this is unlikely, given the magnitude of observed change in fear of childbirth, a randomised controlled trial design would allow evaluation of the impact of the intervention while controlling for relevant factors such as mode of birth.

### 6.4 Recommendations for Further Research

Measuring fear of childbirth with the FOBS scale proved to be feasible. However, before proceeding to a definitive trial, the FOBS must be properly validated for the Finnish setting. Future studies should also examine the cost–benefit aspects of such an intervention, providing insights into the economic impact when delivered as an adjunct to standard care. Due to pragmatic reasons, this study could not include longer‐term participant follow‐up. Future research should incorporate longer‐term follow‐up to assess the future reproductive and perinatal health of the mothers. In the context of this study, conducted in a small central maternity hospital, the number of women screened and meeting the eligibility criteria was relatively small. Therefore, for a future definitive trial, a multicentre randomised approach should be selected.

### 6.5 Implications for Practice

The findings underscore the importance of group‐based support and birthing classes for multiparous women experiencing fear of childbirth, encouraging healthcare providers to implement similar interventions. Additionally, fear of childbirth is a multifaceted phenomenon, and integrating multiprofessional care from psychiatric nursing and midwifery should be promoted to better support women with fear of childbirth.

## 7 Conclusion

The MOTIVE intervention appears to be a feasible, acceptable, and potentially effective approach for reducing fear of childbirth among pregnant multiparous women in a central maternity hospital setting in Finland. Following minor modifications to the intervention, a multicentre randomised controlled pilot trial is warranted to validate these preliminary findings.

## Author Contributions

Made substantial contributions to conception and design, or acquisition of data, or analysis and interpretation of data: L.S., M.K., H.H., A.L.A. Involved in drafting the manuscript or revising it critically for important intellectual content: L.S., M.K., H.H., A.L.A. Given final approval of the version to be published. Each author should have participated sufficiently in the work to take public responsibility for appropriate portions of the content: L.S., M.K., H.H., A.L.A. Agreed to be accountable for all aspects of the work in ensuring that questions related to the accuracy or integrity of any part of the work are appropriately investigated and resolved: L.S., M.K., H.H., A.L.A.

## Ethics Statement

Ethical approval was granted by Pirkanmaa Wellbeing Services County's Regional Research Ethics Committee (R22124H/14.2.2023) and study approval was gained by Kanta‐Häme Central Hospital (HVA/1275/13.00.01/2023).

## Consent

Written consent was sought from individuals who agreed to participate in the study.

## Conflicts of Interest

The authors declare no conflicts of interest.

## Supporting information


**Data S1.** Supplementary file S1.

**Data S2.** Supplementary file S2.

**Data S3.** Supplementary file S3.

## Data Availability

The data that support the findings of this study are available on request from the corresponding author. The data are not publicly available due to privacy or ethical restrictions.
