# Supplementary figures and images for: Evaluating a Group‐Based Intervention Addressing Fear of Childbirth in Multiparous Pregnant Women: A Mixed Methods Feasibility Study
Source: J Adv Nurs. 2025 May 23;82(4):3872–90. doi: 10.1111/jan.17073 (PMC12994669; doi:10.1111/jan.17073)

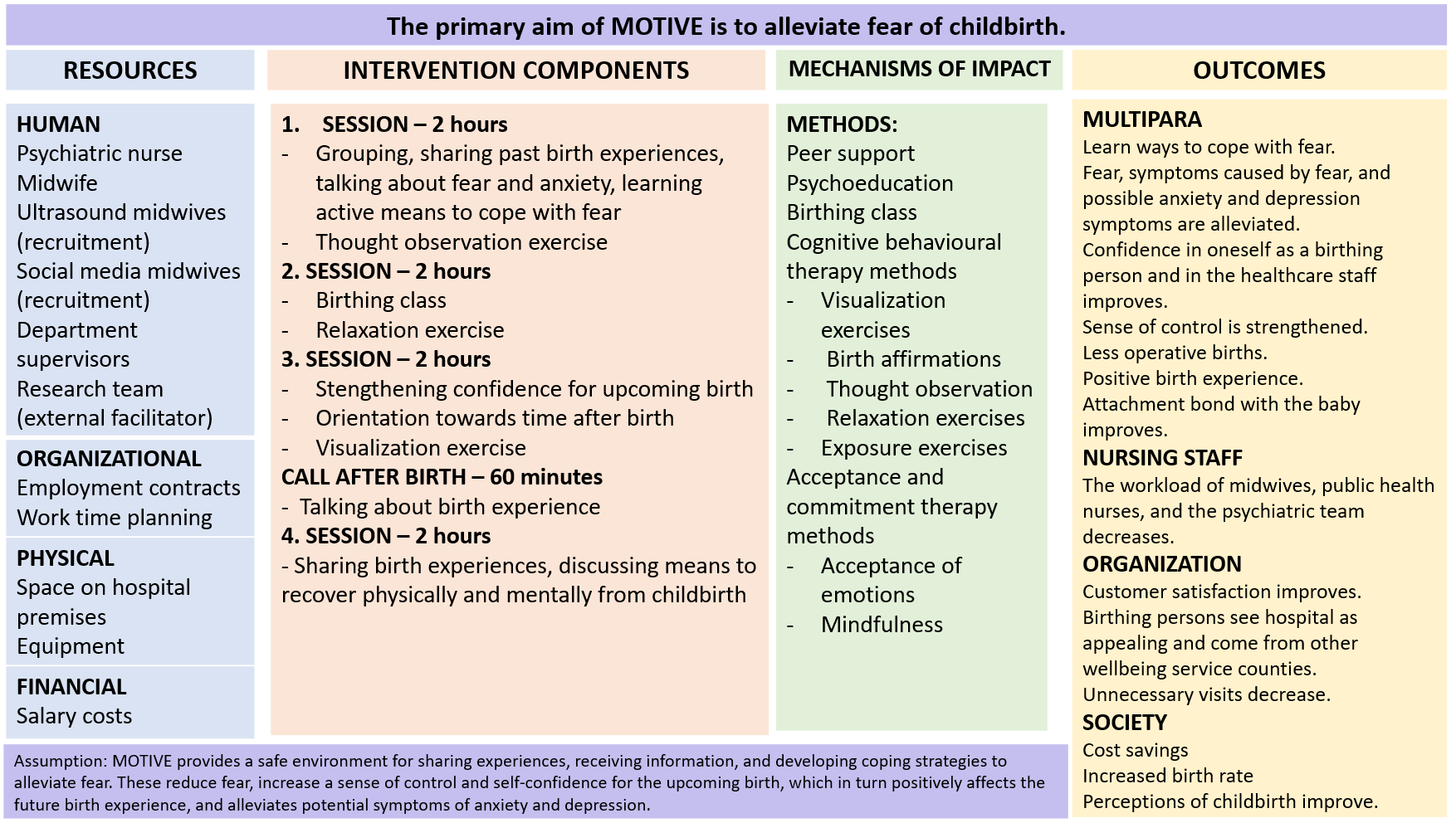


Figure 1. Logic model for MOTIVE

Supplement: Supplementary file 3 — Data S3. Supplementary file S3. [file JAN-82-3872-s002.docx]
